# The multi-modal fusion in visual question answering: a review of attention mechanisms

**DOI:** 10.7717/peerj-cs.1400

**Published:** 2023-05-30

**Authors:** Siyu Lu, Mingzhe Liu, Lirong Yin, Zhengtong Yin, Xuan Liu, Wenfeng Zheng

**Affiliations:** 1 School of Automation Engineering, University of Electronic Science and Technology of China, Chengdu, Sichuan Province, China; 2 School of Data Science and Artificial Intelligence, Wenzhou University of Technology, Wenzhou, China; 3 Department of Geography and Anthropology, Louisiana State University, Baton Rouge, LA, United States of America; 4 College of Resource and Environment Engineering, Guizhou University, Guiyang, China; 5 School of Public Affairs and Administration, University of Electronic Science and Technology of China, Chengdu, China

**Keywords:** VQA, Visual question answering, Multi-modal, Fusion, Attention mechanisms, Attention

## Abstract

Visual Question Answering (VQA) is a significant cross-disciplinary issue in the fields of computer vision and natural language processing that requires a computer to output a natural language answer based on pictures and questions posed based on the pictures. This requires simultaneous processing of multimodal fusion of text features and visual features, and the key task that can ensure its success is the attention mechanism. Bringing in attention mechanisms makes it better to integrate text features and image features into a compact multi-modal representation. Therefore, it is necessary to clarify the development status of attention mechanism, understand the most advanced attention mechanism methods, and look forward to its future development direction. In this article, we first conduct a bibliometric analysis of the correlation through CiteSpace, then we find and reasonably speculate that the attention mechanism has great development potential in cross-modal retrieval. Secondly, we discuss the classification and application of existing attention mechanisms in VQA tasks, analysis their shortcomings, and summarize current improvement methods. Finally, through the continuous exploration of attention mechanisms, we believe that VQA will evolve in a smarter and more human direction.

## Introduction

With the rapid development of deep learning and big data techniques, quantities of remarkable achievements have been made in areas such as computer vision (Ren et al, 2017; Ronneberger, Fischer & Brox, 2015) and natural language processing (Bahdanau, Cho & Bengio, 2015; Luong, Pham & Manning, 2015), These milestones have all enabled the ability to recognize a single modality. However, numerous implementations within the field of artificial intelligence involve multiple modalities. Modality, initially, refers to a biological concept. For example, human beings can receive information from the outside world by virtue of their sensory organs and experiences, including vision, hearing, touch, etc. Each of these forms of information can be called a modality. Multi-modal interaction refers to the integration of information from various senses, thus making it easy for people to communicate with the outside world.

Image caption task (Anderson et al., 2018; Chen et al., 2017; Xu et al., 2015) first combines computer vision with natural language processing. Similarly, VQA is a dynamic multidisciplinary field, which attracts increasing interest and has extraordinary potential (Wu et al., 2017; Antol et al., 2015). Given a picture and a natural language question including which, when, where, who, what, how and why (Zhu et al., 2016) that is correlative with this picture, the system need to produce a natural language answer as an output. It is clear that this is a typical difficult multi-modal task that combines computer vision (CV) and natural language processing (NLP) as two major techniques, and its main objective is for the computer to produce an answer that complies with natural language rules and has reasonable content based on the input image and question. In this task, computers need to understand and integrate information from multiple modalities, such as text features and image features, and build models that can deal with and correlate information from multiple modalities, which is obviously very challenging. It has a wide range of applications and is crucial in many real-world situations, such as medical research (Zheng et al., 2020; Pan et al., 2022; Li et al., 2022; Gong et al., 2021; Zhan et al., 2020; Wang et al., 2022a; Wang et al., 2022b), remote sensing (Bazi et al., 2022; Zheng et al., 2022b; Al Rahhal et al., 2022), and blindness help (Gurari et al., 2018; Tung et al., 2021; Tung, Huy Tien & Minh Le, 2021). Therefore, it is of great significance to improve existing VQA methods.

The algorithms in visual question answering can be broadly divided into three steps: extracting features from images, extracting features from questions, and finally combining image and text features to generate answers. The third of these steps, which is how to better fuse image features with text features, is the core problem of the VQA task (Liu et al., 2019; Gao et al., 2019). As a fundamental multi-modal task, VQA requires a comprehension of both the problem’s constituent structure and the complex substance in the image, and should also have the ability to correctly extract and integrate information from both the image and the text, that’s what makes it so challenging. Therefore, the study of cross-modal modeling methods is of broad interest, and how to better integrate text features and image feature sets into a compact multi-modal representation has become an important research topic.

**Figure 1:**
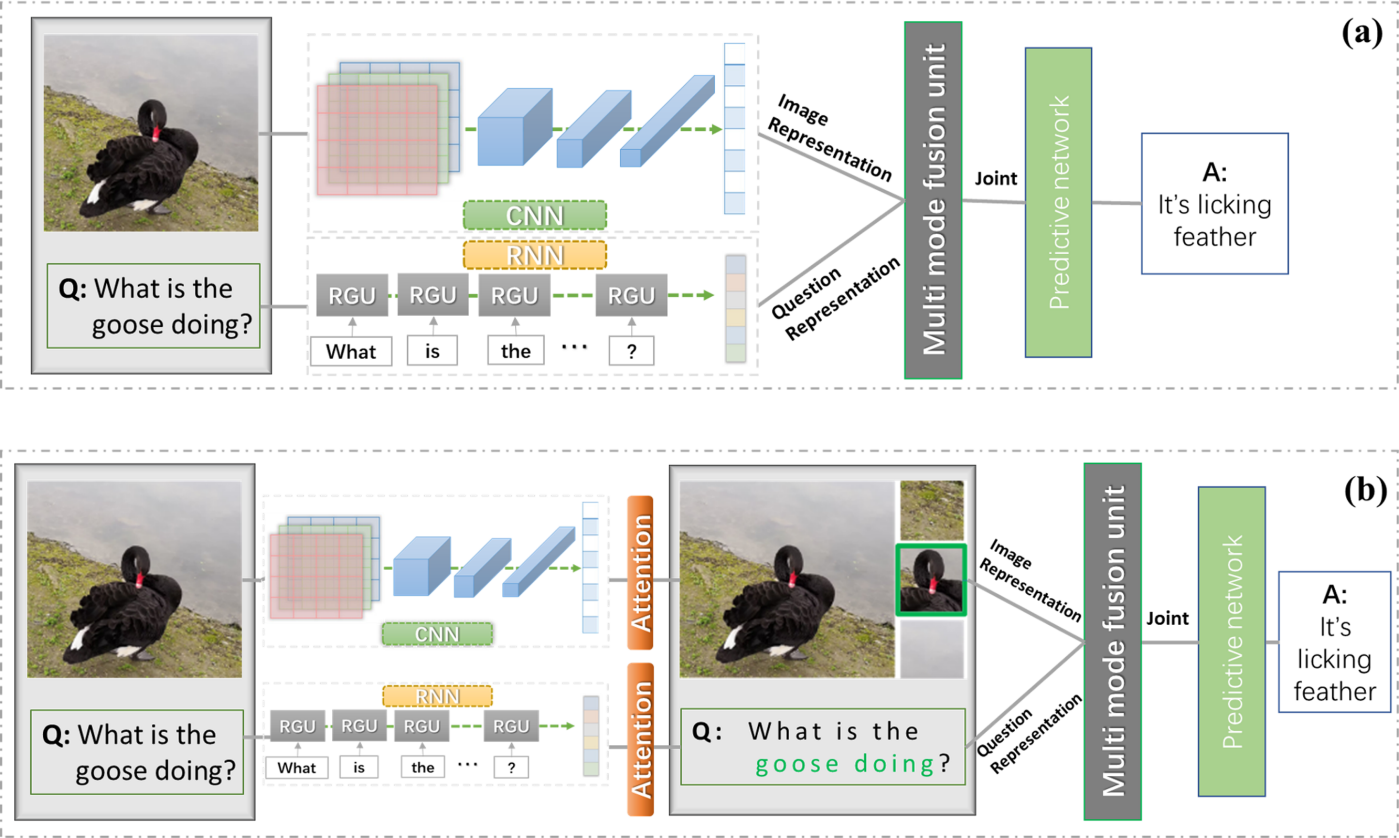
VQA task with/without attention mechanism.

In recent studies, the difference in algorithms was mainly in how to combine the features of both to do the processing. Most of the existing methods focus on learning the joint representation of image and question. Under the framework of such methods, question and image are usually encoded with RNN (LSTM most commonly used) and CNN (Selvaraju et al., 2020; Gao et al., 2015; Malinowski et al., 2015), respectively, and then their representation vectors are input into the multimode fusion unit. The aligned joint representation vector is obtained by training the fusion unit, and finally the representation vector is input into a neural network to get the final answer, as shown in Fig. 1A. However, this is actually a simple and straightforward combination of features that cannot correctly discriminate what is really useful information, the concerns are all global in character, making it difficult to correctly infer the answer and greatly limiting the performance of VQA. Therefore, in order to make VQA models better grasp the problem and identify the key visual content, scholars are now more committed to studying attention mechanisms.

It can be said that attention models have become almost ubiquitous in deep neural networks. The attention mechanism is inspired by the human attention mechanism, in which people tend to selectively observe and pay attention to specific parts of information while ignoring the rest, as needed. That is, the observation can be adjusted to the more informative features according to their relative importance, focusing the algorithm on the most relevant parts of the input, moving from focusing on global features to the focused features, thus saving resources and getting the most effective information quickly. The attention mechanism has arguably become one of the most important concepts in the field of deep learning, since Bahdanau, Cho & Bengio (2015) used attention mechanism for the machine interpretation tasks, various variants of attention mechanism have emerged, such as Co-Attention networks (Yang et al., 2019a; Han et al., 2021; Yu et al., 2019; Liu et al., 2021b; Lu et al., 2016; Sharma & Srivastava, 2022), Recurrent Attention networks (Osman & Samek, 2019; Ren & Zemel, 2017; Gan et al., 2019), Self-Attention networks (Li et al., 2019; Fan et al., 2019; Ramachandran et al., 2019; Xia et al., 2022; Xiang et al., 2022; Yan, Silamu & Li, 2022), *etc.* The effectiveness of visual information processing is considerably enhanced by all of these attention mechanisms, which also optimize VQA performance.

Its advantages can be summarized in two simple points:

(1) Improvement of computational power: Because of remembering a large amount of information, the model becomes complex, and at present, the computational power remains an important factor limiting the neural network.

(2) Simplification of the model: It can make the neural network a bit simpler and effectively alleviate the contradiction between model complexity and expressive power.

While most previous work has reviewed the entire field of VQA, the focal point of this article is to give an overview of “attention mechanisms” in multi-modal fusion methods for VQA tasks. Nowadays, with the rapid development of information technology, multi-modal data has become the main form of data resource in recent years. Therefore, the study of multi-modal learning development can empower computers to understand diverse data, so this research topic has an important value: how to better utilize the information of multiple modalities is of great significance to the study of deep learning. At the same time, multi-modal data can also provide more information for model decision making, thus improving the overall accuracy of the decision. A thorough research review can be produced through the analysis and evaluation of its existing research. This article can help pertinent researchers to have a more comprehensive understanding of the attention mechanism in multimodal fusion methods.

The study is organized as follows: in Section 2, we describe how we comprehensively collect relevant literature and provide a comprehensive analysis of VQA using bibliometric methods, including analysis of keywords, countries and institutions, and timelines; in Section 3, We describe the general model of attention mechanism, and classify the attention mechanisms commonly used in VQA tasks from three dimensions; in Section 4, We describe the application of attention mechanism from two aspects; in Section 5, we analyzed and compared the attention mechanism used by some classical frontier models, and summarized the development trend of attention mechanism in VQA; in Section 6, we conclude the article and prospect the possible future directions.

## Survey Methodology

Bibliometric methods are used to comprehensively analyze the current state of attention mechanisms in VQA and conclude the article with a prospect of possible future directions. A total of 420 eligible articles in the database of Web of Science (SCIE only, since 2011) with “Visual Question Answering” and “attention mechanism” as the subject line were retrieved and screened. The retrieved articles were saved as plain text files, counting full records and cited references, and then we used the “Data/Import/Export” function of CiteSpace (Rawat & Sood, 2021) to convert them into an executable format and visualize the retrieved records. A visual analysis of the collected literature has been made. We selected the top 50 levels and 10.0% of most cited or occurred items from each slice, and tick at minimum spanning tree in pruning. This study aims to systematically review the development history and research status of VQA and attention mechanism with a bibliometric approach, explore the hotspots in the field, and reasonably predict the possible future directions.

### Analysis of keywords

CiteSpace is used to perform keyword co-occurrence analysis on the above articles, setting the time slice as January 2010 to December 2022, one year per slice, and choosing “keyword” for the node type. The figure shows keywords with more than seven occurrences.

The size of the nodes and labels indicates the frequency of the keyword. From the figure, it can be found that “Visual Question Answering”(120), “attention mechanisms”(43), “deep learning”(35), “task analysis”(34), “video question answering”(25), “knowledge discovery” (24) “computer vision”(20) appeared most frequently.

**Figure 2:**
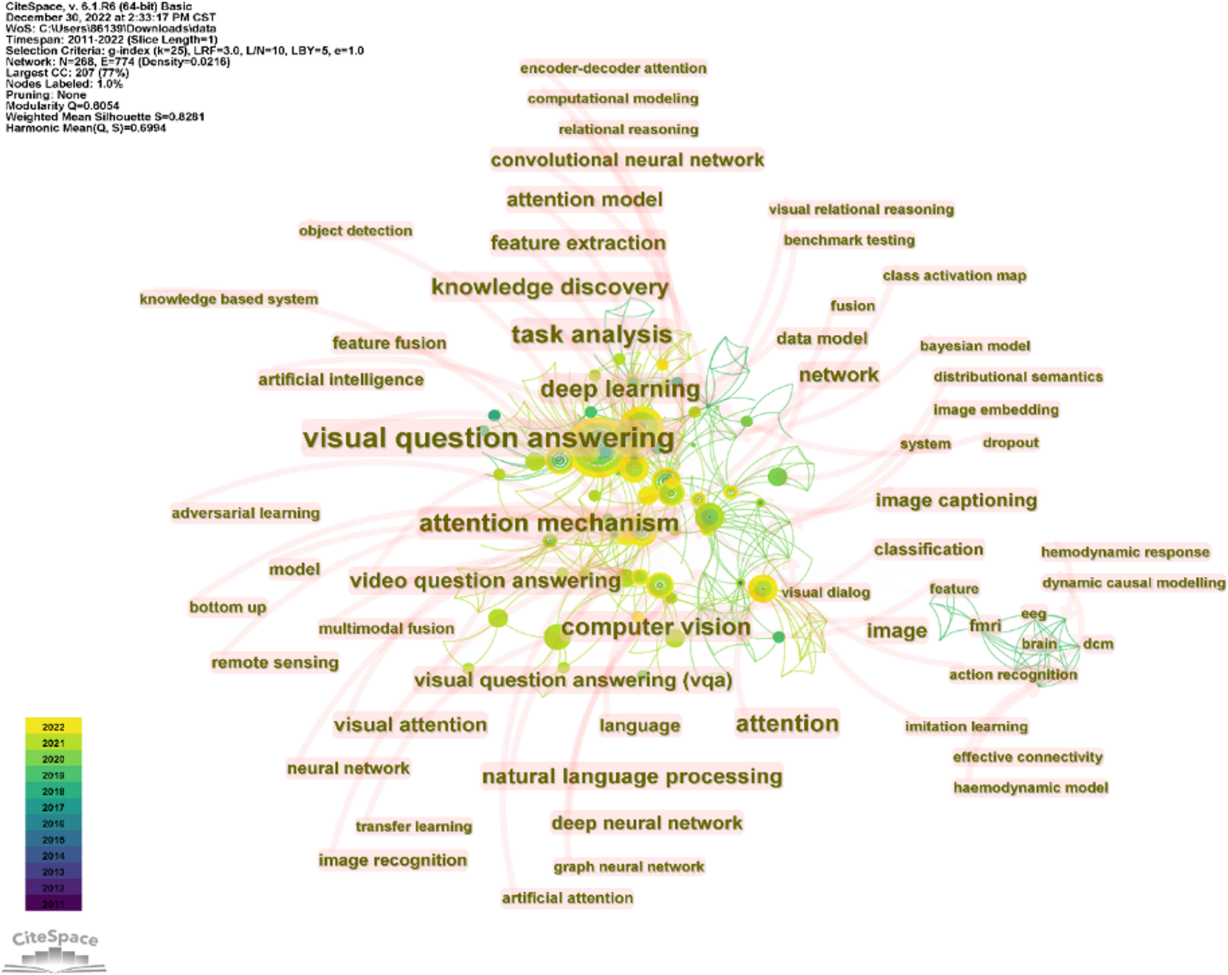
Keywords co-occurrence.

The cluster view tells us the current research frontier topics. From the Fig. 2, we can infer that attention mechanisms, semantic analysis, visual question answering, and multi-modal learning are the current research hotspots.

### Analysis of leading collaborating countries and institution

**Figure 3:**
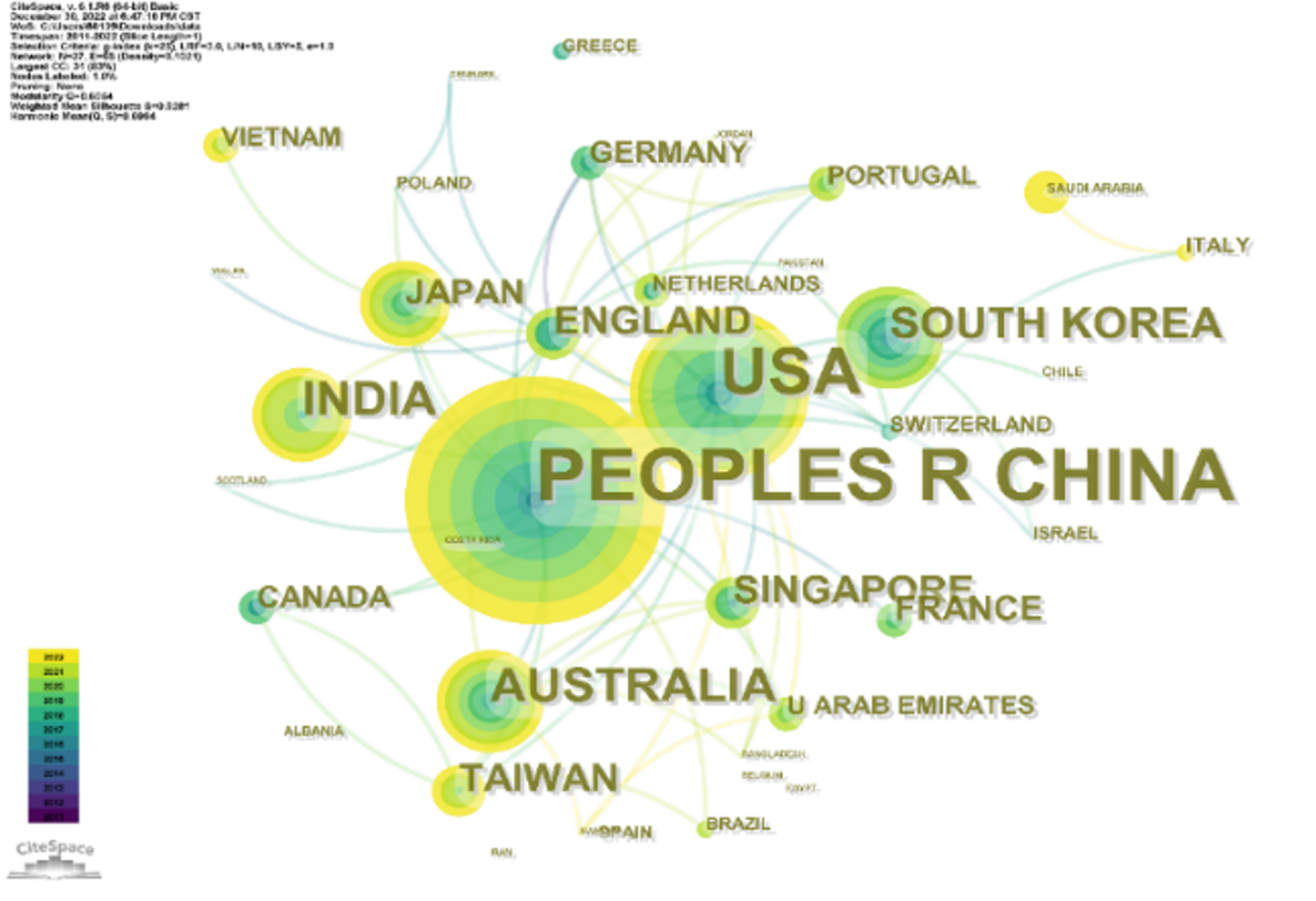
Leading collaborating countries.

**Figure 4:**
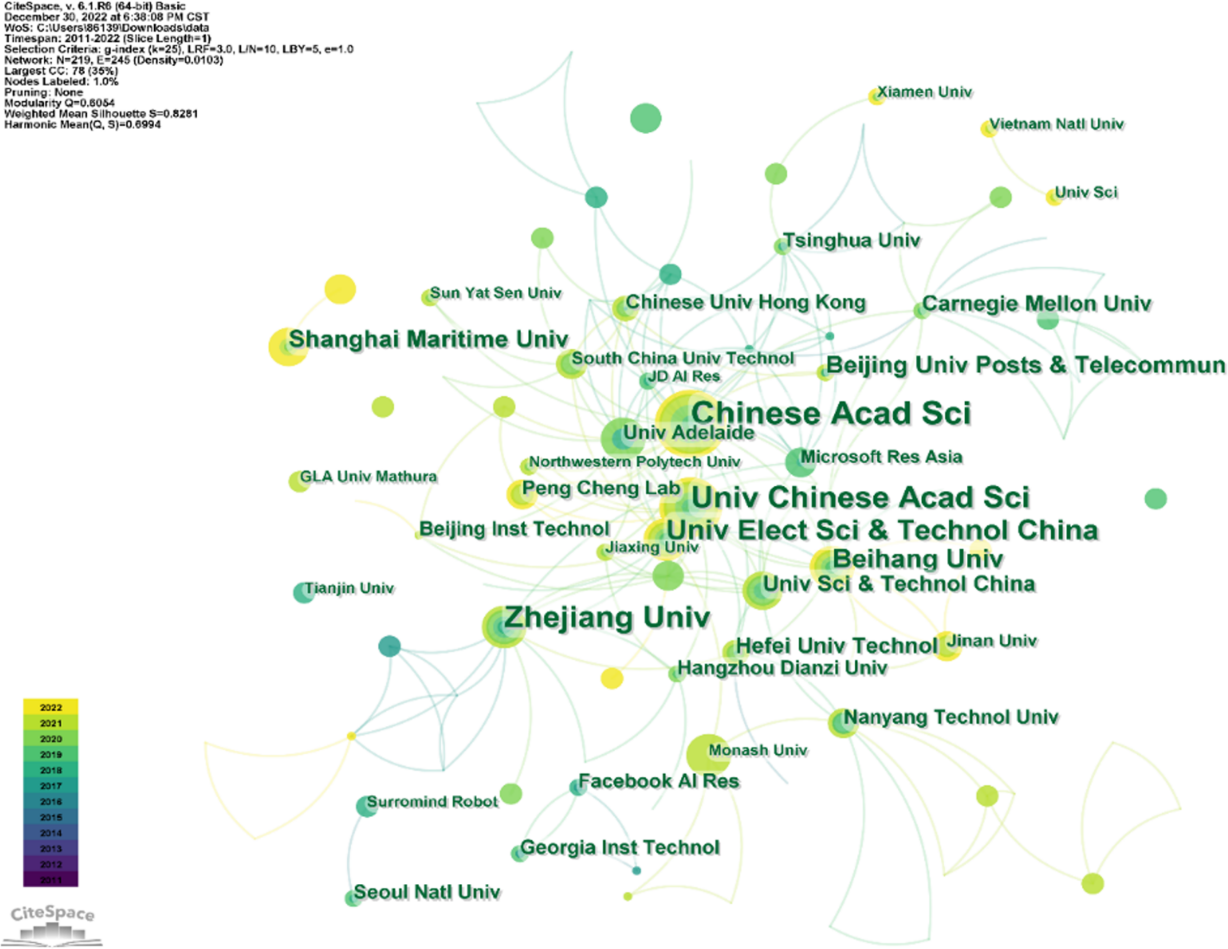
Leading collaborating institutions.

From 2010 to 2022, research institutions from 37 countries are all making irreplaceable contributions in this field. Figures 3 and 4 show the visualization of these countries and institutions.

In Fig. 3 there are a total of 37 nodes, and the larger the area of the circle of the node, the greater the number of publications from that country, with the color indicating the year of citation. As we can see from the figure, the five countries that contribute the most regarding the number of distributions are China, the United States, India, Australia, and South Korea. Undoubtedly, Chinese researchers have played an important role in the research in this field, with 242 publications by Chinese scholars accounting for 57.619% of the total number of publications (420). The United States ranks second, with 100 articles contributing to the field, accounting for 23.810%. In addition, as an example of emerging research countries, India, Australia and South Korea have also made significant contributions in the last decade. Australia ranked third with 7.143%. Other high-producing countries include Australia (5.714%), South Korea (4.762%), and others.

As we can see in Fig. 4, the five institutions that contribute the most to the number of publications are Chinese Academy of Science, Zhejiang University, University of the Chinese Academy of Sciences, Zhejiang University, University of Electronic Science and Technology of China, and Beihang University, which also shows once again that Chinese researchers are playing an irreplaceable role in research in this field.

China is the most populous country in the world, and there are many universities and research institutions. At present, AI is developing rapidly. As one of the applications of intelligent systems, VQA has attracted more Chinese researchers and scientists, focusing on this frontier field and promoting research progress in related fields (Guo & Han, 2022; Guo & Han, 2023; Miao et al., 2022b; Miao et al., 2022a; Peng et al., 2022a; Shen et al., 2022; Liu et al., 2022a).

### Analysis of timeline

**Figure 5:**
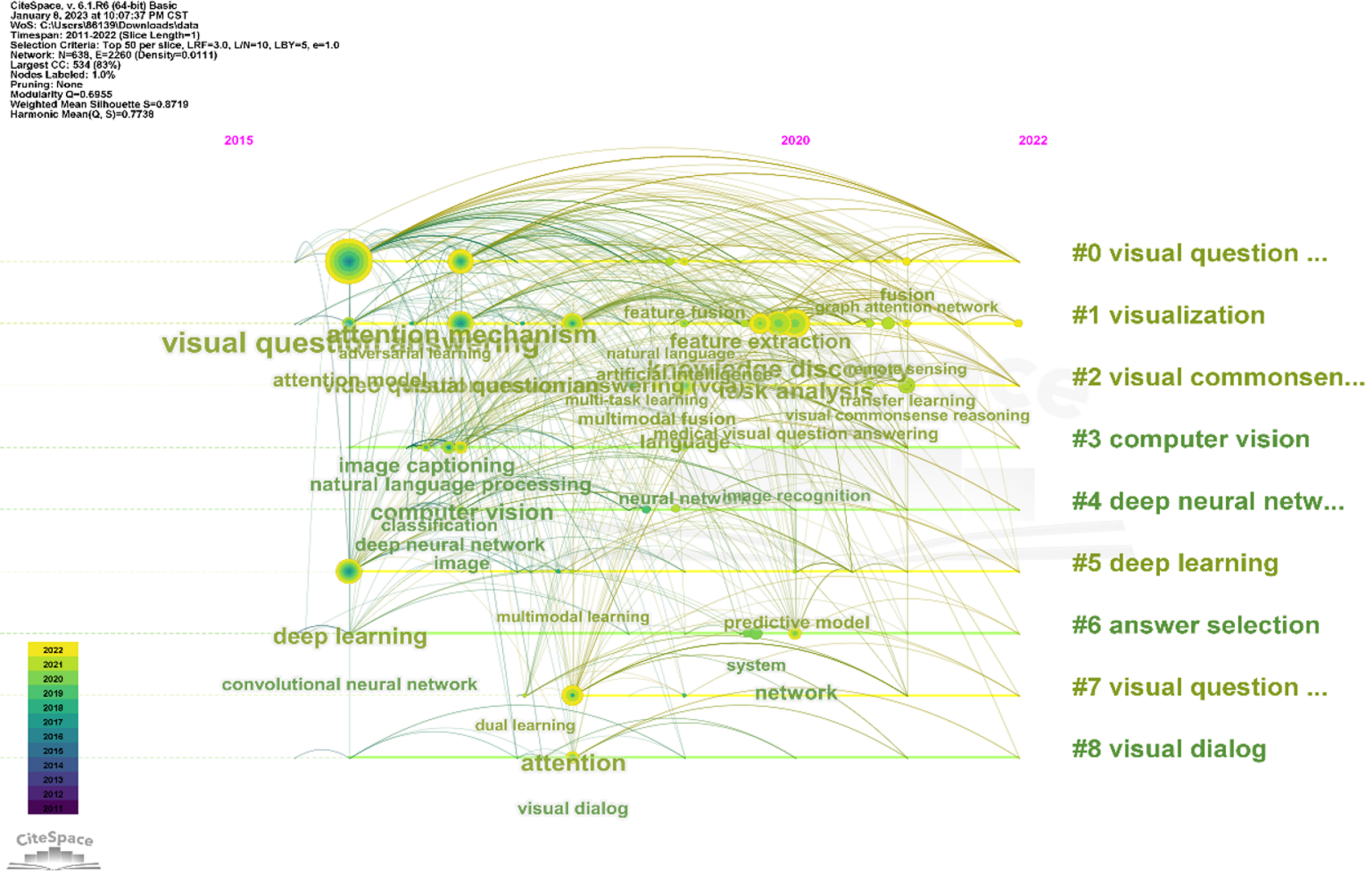
Timeline of keywords clusters.

Figure 5 shows the timeline of keywords. Table 1 shows the clustering result of keywords. Each line represents a cluster, and every single line from shows the evolvement of keywords over time. Words like “visual question answering”, “attention module” and “deep learning” appears in 2016. This shows that researchers began to apply attention mechanism to VQA tasks gradually in 2016. So far, we have screened a total of 420 articles, indicating that the current research in this area is not saturated, and there are still many methods to be explored. In 2020, nodes have increased a lot in almost every time line, indicating that research in this field has been developing rapidly since 2020. As the largest node in 2020, knowledge discovery may become a research hotspot in the future. It can be seen that over time, people’s research in this field has been gradually subdivided and deepened, and more models and methods have been applied to solve the problem of VQA. VQA research has also penetrated into different aspects, such as medical and remote sensing.

**Table 1:** Keyword cluster analysis.

Clusters	Ranked terms
#0: visual question answering	parameter-sharing mechanism; local perception; top-down attention; cascading top-down attention — attention mechanism; relational reasoning; neural network; graph convolution; sematic relationship visual; question; answering; mechanism; bilinear attention; vision; answerability; transformer; multi-head
#1: knowledge discovery	task analysis; knowledge discovery; feature extraction; visual question; adversarial learning — visual question answering; attention model; language parsing; deep reasoning; image coding mechanism; sensing; remote; object; understanding graph; deep; bilinear; gnns; modeling
#2: video question answering	video question answering; multi-head attention; referring expression generation; knowledge discovery; multi-level feature fusion — medical visual question answering; transfer learning; data augmentation; abnormality questions; global average feature; character; multi-level; interaction; optical learning; data; modality; multi-task; planes
#3: Computer vision	Computer vision; visual question answering; sparse question self-attention; dual self-guided attention; artificial attention — natural language processing; deep learning; dense co-attention network; distributional semantics; image captioning question; attention; self-guided; sparse; self-attention semantics; knowledge; analysis; task; machine
#4: deep neural networks	deep neural networks; attention mechanisms; age recognition; gender recognition; neural network — neuromorphic computing; deep learning; artificial neural networks; spiking neural networks; simulated annealing bidirectional; literature; convolutional; transformers; self-supervision spiking; learning; neuromorphic; computing; benchmark
#5: deep learning	deep learning; visual question answering; short-term memory; mood detection; attention model — task analysis; visual question; prediction algorithms; relational reasoning; graph matching attention detection; memory; long; short-term; mood feature; description; audio; encoder–decoder; soundnet
#6: dynamic causal modelling	answer selection; attention mechanism; convolutional neural network; bidirectional lstm; siamese network — text analysis; feature construction; answer recommendation; community question answering; feature encoding answer; feature; answering; construction; biologically semantic; common; hierarchical; compositionality; knowledge
#7: dual learning	dual learning; visual question answer; policy gradient; bidirectional encoder representation; question popularity — visual question generation; attention mechanism; reasoning models; visual question answering; reinforcement learning knowledge; diffusion; network; popularity; forum bidirectional; representation; feature; multi-modal; transformers
#8: visual dialog	visual dialog; attention network; visual reference; multimodal semantic interaction; textual reference — question answering; focal attention; photo albums; vision-language understanding; heterogeneous information visual; reference; multimodal; textual; interaction spectral; reasoning; simple; convolution; dual-perspective

## Classification of Attention Mechanisms in VQA

In this chapter, we describe the general mechanism of attention and categorize attention by scope, excitation, and dimension.

### General attention mechanism

From the mathematical perspective, the attention mechanism simply assigns different weighting parameters to input items according to their importance, but this mechanism simulates the cognitive model of the human brain, that is, focusing limited attention on the key parts of things according to actual needs, thus greatly enhancing the understanding ability of neural networks.

**Figure 6:**
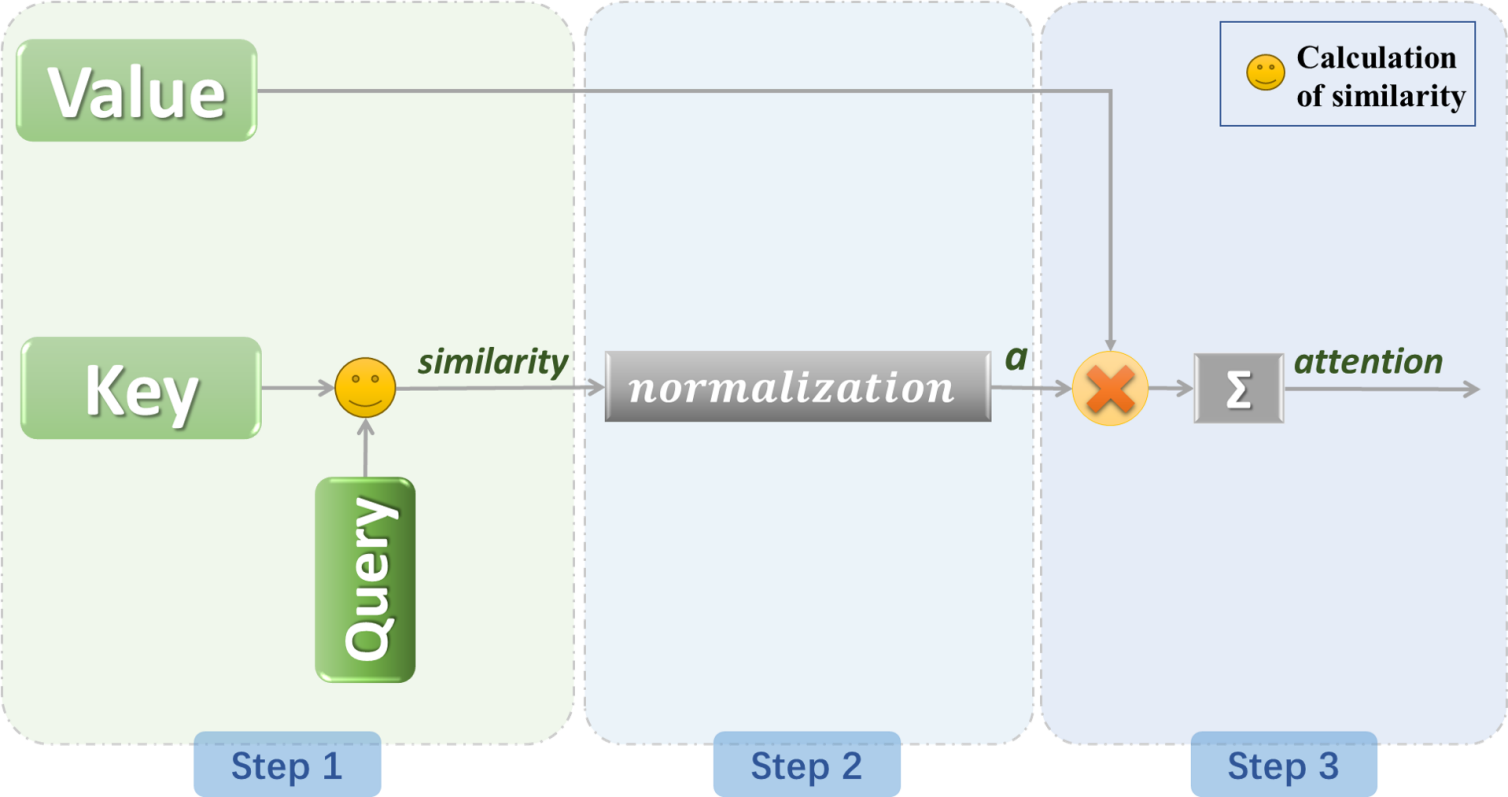
General model of attention mechanism.

Suppose that the constituent elements in the source are composed of a series of key value data pairs. At this time, given an element Query in the target, calculate the similarity or correlation between Query and each key to obtain the weight coefficient of the corresponding value of each key, and then add weights to sum the values to obtain the final attention value (Vaswani et al., 2017). Therefore, essentially, the attention mechanism is a weighted sum of the value values of the elements in the source, while Query and Key are used to calculate the weight coefficients of the corresponding values. The keys and values are also packed into K and V. The calculation of attention is generally divided into 3 steps (Chaudhari et al., 2021), as shown in Fig. 6.

(1) The first step is to calculate the similarity between Query and different keys, that is, to calculate the weight coefficients of different Value values; There are mainly three ways to calculate similarity.

(1) \begin{eqnarray*}{\text{Similarity}}_{\text{Dotproduct}} \left( \mathrm{Q},\mathrm{K} \right) & =\mathrm{Q}\cdot \mathrm{K}\end{eqnarray*}

(2) \begin{eqnarray*}{\text{Similarity}}_{Cosine}(\mathrm{Q},\mathrm{K})& = \frac{\mathrm{Q}\cdot \mathrm{K}}{\parallel \mathrm{Q}\parallel \cdot \parallel \mathrm{K}\parallel } \end{eqnarray*}

(3) \begin{eqnarray*}{\text{Similarity}}_{MLP} \left( \mathrm{Q},\mathrm{K} \right) & =MLP(\mathrm{Q}\cdot \mathrm{K})\end{eqnarray*}

(2) In the second step, the output of the previous stage is normalized to map the range of values between 0 and 1. The most commonly used is softmax.

(4) \begin{eqnarray*}{a}_{i}=\text{softmax} \left( {\text{Similarity}}_{\mathrm{}i} \right) = \frac{{e}^{{\text{Sim}}_{i}}}{\sum _{j=1}^{Lx}\,{e}^{{\text{Sim}}_{j}}} \end{eqnarray*}

*a*_*i*_ indicates the degree of attention paid to the ith information.

(3) In the third step, accumulate the result by multiplying the value and the weight corresponding to each value to obtain the attention value.

(5) \begin{eqnarray*}\text{Attention}~(\text{Query},\text{Source})=\sum _{i=1}^{Lx}\,{a}_{i}\cdot {\mathrm{V }}_{i}\end{eqnarray*}

### Classification of attention mechanisms

**Figure 7:**
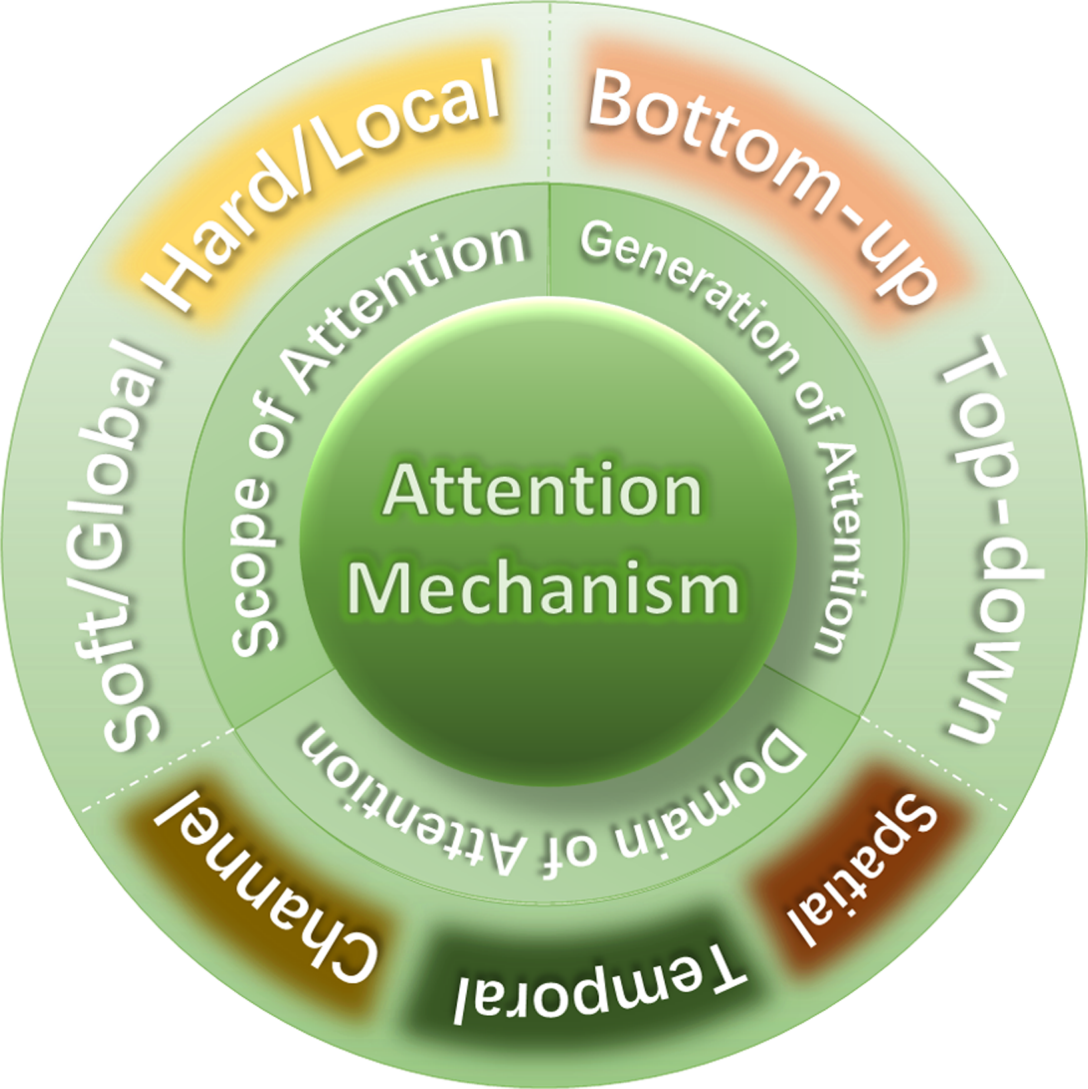
Classification of attention mechanism.

We have classified the attention mechanism into three aspects, as shown in Fig. 7.

#### Scope of attention

According to the scope of attention, attention can be divided into soft attention and hard attention, or local attention and global attention.

Soft and hard attention were proposed by Xu et al. (2015) in an image caption task. The soft attention will focus on all the positions of the. Soft attention refers to when selecting information, instead of selecting only one of the N information, it calculates the weighted average of the N input information. For image (Niu, Zhong & Yu, 2021), it means giving different weights to different positions and then inputting it into the neural network for calculation. This is also similar to Bahdanau attention (Bahdanau, Cho & Bengio, 2015).

However, in hard attention, only one image block is considered at a time (Malinowski et al., 2018). Soft attention pays more attention to the overall information, which can be differentiated and expensive. Hard attention only focuses on a certain part of the information, which is not differentiable and is not expensive to calculate.

Global attention and local attention were proposed by Luong, Pham & Manning (2015) for a language translation task.

The initial global attention defined by Luong, Pham & Manning (2015) considers all the hidden states of the encoder LSTM (Cheng, Dong & Lapata, 2016) and decoder LSTM to calculate the “variable length context vector”, while Bahdanau, Cho & Bengio (2015) use the previous hidden states of the unidirectional decoder LSTM and all the hidden states of the encoder LSTM to calculate the context vector. When applying the “global” attention layer, a lot of calculations will be generated. This is because all hidden states must be considered.

Local attention is a mixture of soft attention and hard attention. It does not consider all coded inputs, but only a part of the inputs. This not only avoids the expensive computation caused by soft attention, but also is easier to train than hard attention. Compared with global attention, local attention is much less computationally expensive.

#### Generation of attention

According to the generation of attention, attention can be divided into bottom-up attention and top-down attention.

Researchers apply hard attention to image caption as bottom-up attention (Anderson et al., 2018; Teney et al., 2018). The term top-down and bottom-up attention was first proposed in neurological articles. Anderson integrated top down and bottom-up attention and presented a new model of visual question and answer (Luong, Pham & Manning, 2015), referring to attention mechanisms driven by non-visual or task-specific context as ‘top-down’, and purely visual feed-forward attention mechanisms as ‘bottom-up’. Bottom up is also called focal attention (Liang et al., 2019; Liang et al., 2018).

The bottom up attention in Anderson et al. (2018) is implemented by object detection network Faster R-CNN (Ren et al., 2017), which divides the image into specific objects for filtering. Annotate specific elements in a given image by using the Visual Genome dataset. The resulting features can be interpreted as ResNet features centered on the first K objects in the image. That is to say, instead of using the whole picture as the visual feature, the proposal in the first K pictures is selected as the visual feature.

The top-down attention, that is, the problem feature is that after the top is concatenated with the features of each proposal, an attention is obtained through the nonlinear layer and the linear layer, and then the attention is multiplied with the visual feature to get a better feature. Top-down attention is to determine the contribution of features to the text.

#### Domain of attention

According to the dimension of attention, attention can be divided into spatial (Yan et al., 2022), channel and temporal attention (Guo et al., 2022) etc.

Spatial attention can be regarded as an adaptive spatial region selection mechanism and answers the question of “where”. Channel attention adaptively recalibrates the weight of each channel, which can be regarded as an object selection process and answers the question of “what” or “which”. As the name indicates, temporal attention can be used to answer the question of “when”.

The existing VQA model usually focuses on the spatial dimension. Considering that attribute features are as important as the area of interest, Zhang et al. (2021) constructed an effective joint feature and spatial common attention network (UFSCAN) model for VQA.

The general Spatial visual attention only selects the most concerned visual regions and uses the same weight on the channel, which does not conform to the idea of attention (Hu et al., 2020). CNN is naturally characterized by space and channel. In addition, the visual attention model is usually performed at the pixel level, which may lead to the problem of regional discontinuity. Song et al. (2018) proposed a cubic visual attention to select important information and improved VQA tasks by successfully applying new channel and spatial attention to object regions.

For the Question Answering video (Kossi et al., 2022; Liu et al., 2022b; Suresh et al., 2022; Varga, 2022), which needs to pay attention to specific frames, or visual dialog (Guo, Wang & Wang, 2019; Kang et al., 2019; Patro, Anupriy & Namboodiri, 2022), which should be able to capture a temporary context from a dialog history, temporal attention is needed (Yang et al., 2019b; Seo et al., 2017). The existing attention mechanism of VQA mainly focuses on the attention in the spatial area of the image or in a single sequence, so it may not make full use of the properties of multiple sequences and multiple time steps (Zhao et al., 2017; Jiang et al., 2020; Chen et al., 2019; Shi et al., 2018). Combining spatial and temporal attention, an encoder decoder model is proposed by Zhao et al. (2017) to solve the open video question answering problem.

## Application of Attention Mechanisms in VQA

**Figure 8:**
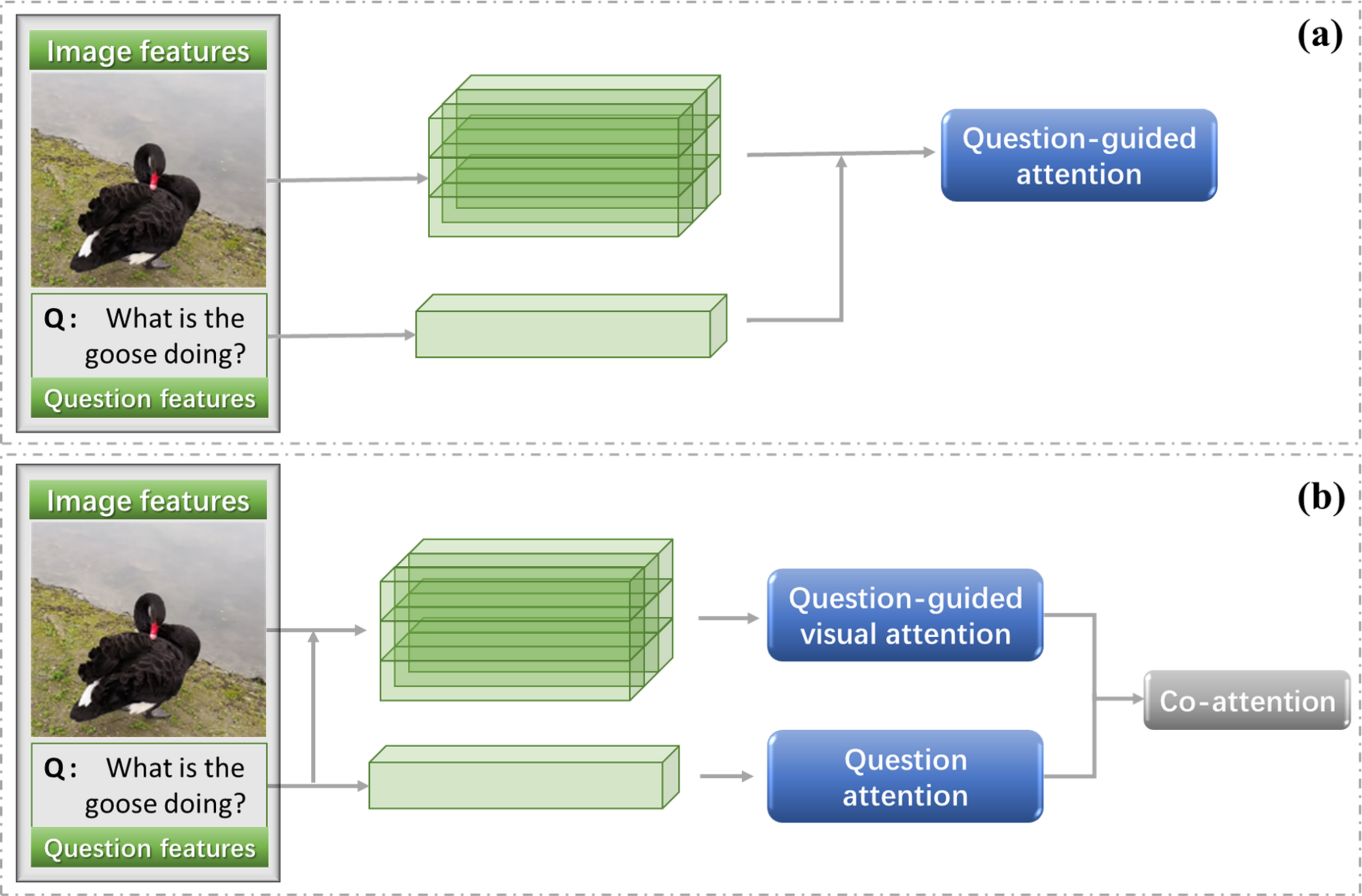
Question-guided attention (A) and co-attention (B).

In this article, we divide the explanation of attention mechanism into problem-directed attention and joint attention, as shown in Fig. 8.

### Question-guided visual attention

The initial attention mechanism in VQA tasks considers how to use the question to find the related area in the image. The general practice is to assign weights to image regions according to their relevance to a question (Liu et al., 2022a; Chen et al., 2015). Figure 8 shows the general model of question guided image attention. The image regions can be divided according to the location or object detection box.

#### Attention learned from free form regions

A stacking attention network was proposed by Yang et al. (2016) that searches for areas in a picture that are related to the response. It uses semantic representations of questions as queries through several iterations. This is the first time an attention mechanism has been added to a VQA task and the results are encouraging and it is a typical spatial attention method. Since then, researchers have focused more and more on employing attention models to identify significant images for efficient response inference. To a large extent, it tends to rely on strong language priority in training questions. The performance on the test image pair is significantly reduced. GVQA (Agrawal et al., 2018) is based on SAN and contains inductive bias and restriction in structure, which enables the model to more effectively summarize the answers of different distributions.

Li, Yuan & Lu (2021) proposed a dual-level attention network, using the problem guided attention network to select the image area related to the question, using semantics to highlight the concept related area. More relevant spatial information can be found through the combination of two kinds of attention, and thus reducing the semantic gap between vision and language.

The existing attention research answers questions by focusing on a certain image area, but it is believed that the area of attention mechanism of the existing research is not related to the image area that people will pay attention to Das et al. (2017); Qiao et al. (2018). Therefore, Patro & Namboodiri (2018) proposes to obtain a differential attention region through one or more supporting and opposing paradigms. Compared with the image-based attention method, the differential attention calculated in Patro & Namboodiri (2018) is closer to human attention.

#### Attention learned from bounding boxes

The research mentioned in this section use the bounding boxes to select specific objects in the picture, so as to analyze the relationship between different objects (Ilievski, Yan & Feng, 2016; Zhang et al., 2020; Zhu et al., 2021; Yu et al., 2020; Xi et al., 2020).

For example, although the model can detect objects, backgrounds, *etc*. in the image, it is difficult to understand the semantic information about positions and actions. In order to capture the motion and position information of objects in the image, the model needs to have a more comprehensive understanding of the visual scene in the image by analyzing the dynamic interaction between different objects in the image, not just object detection. One possible method is to align the relative geometric position of objects in the image (for example, the motorcycle is beside the car) with the spatial description information in the text. The other is to capture the dynamic interaction in the visual scene by learning the semantic dependency between objects. Based on this, Wu et al. (2018a) introduced a relational attention model. He compares different objects in the image through difference calculation and uses the attention mechanism to filter. Peng et al. (2019) devised two question-adaptive relation attention modules that can extract not only the fine-grained and precise binary relations but also the more sophisticated trinary relations.

We discussed the visual attention mechanisms based on the free form region and bounding boxes respectively above. But sometimes these two attention mechanisms can provide complementary information and should be effectively integrated to better solve VQA problems.

Lu et al. (2018a) proposed a new deep neural network for VQA, which integrates two attention mechanisms. The multi-mode multiplicative feature embedding effectively fuses the features of free form image area, detection frame and question representation, so as to answer questions more accurately.

### Co-attention mechanisms

**Figure 9:**
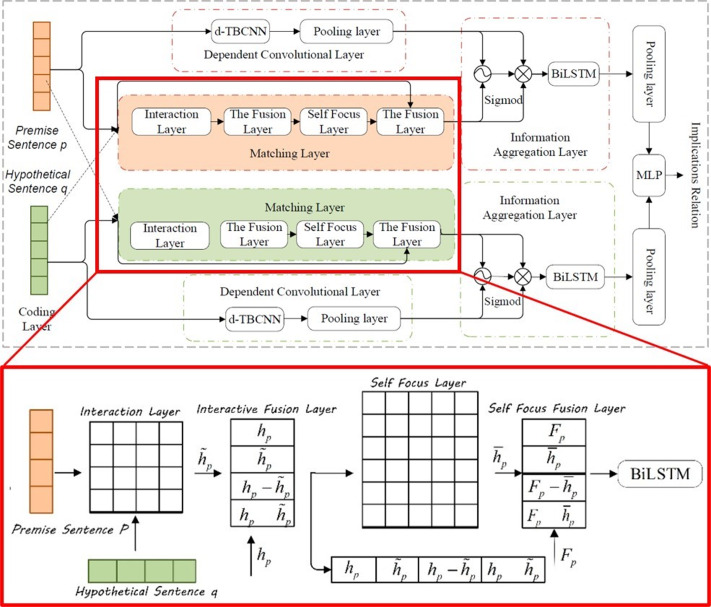
The structure of SCF-DMN model and the network structure of the matching layer (Zheng et al., 2022a).

In the visual question answering task, if the model wants to correctly answer complex questions, it must have a full understanding of the visual scene in the image, especially the interaction between different objects. Although this kind of method can deal with some VQA tasks, it still cannot solve the semantic gap between image and question, as shown in Fig. 9. The attention for question also matters (Burns et al., 2019; Nam, Ha & Kim, 2017; Yu et al., 2018).

Kim, Jun & Zhang (2018) proposes the BAN network to use visual and linguistic information as much as possible. BAN uses bilinear interaction in the group of two input channels, and the low rank bilinear pooling extracts the joint representation of the two channels. In addition, the author also proposes a variant of multimodal residual network to make full use of the attention map of BAN network.

Osman & Samek (2019) proposed a recurrent attention mechanism, which uses dual (textual and visual) recurrent attention Units (RAUs). With this model, the researchers demonstrated the impact of all potential combinations of recurrent and convolutional dual attention. Lu et al. (2018b) proposed a novel sequential attention mechanism to seamlessly combine visual and semantic clues for VQA.

Gao et al. (2019) proposed a new framework (DFAF) for VQA, which dynamically fuses attention flow within and between modes. DFAF frames alternately transfer information within or across modes according to the flow of attention between and within modes.

Lu et al. (2016) proposed a new concept of collaborative saliency of joint image and text features, which enables two features of different modes to guide each other. In addition, the author also weights the input text information from word level, phrase level and question level to build multiple image question co expression maps at different levels.

Nguyen & Okatani (2018) proposes a new cooperative attention mechanism to improve the fusion of visual and linguistic representation. Given the representation of the image and the question, first generate the attention map on the image area for each question word, and generate the attention map on the problem word for each image area.

The attention mechanism has been improved through self-attention. Typically, self-

attention is used as a spatial attention mechanism to capture global information. It is simpler for the model to capture long-distance interdependent elements in a sentence after the introduction of self-attention (Vaswani et al., 2017). Because in the case of RNN or LSTM, it requires sequential sequence computation, and for long-distance interdependent features, linking the two requires multiple time steps of information gathering, and the greater the separation, the more difficult it is to successfully capture them. The distance between long-distance dependent features is greatly reduced, however, because of self-attention, which is an attention mechanism that connects elements at various positions of a single sequence and directly connects the connection of any two words in a sentence through one computation step directly during the computation. As a result, it lessens the reliance on external information and is better at capturing the internal relevance of data or features, which can affect the distance between long-distance dependent features (Chen, Han & Wang, 2020; Khan et al., 2022). In addition, self-attention is also helpful for increasing the parallelism of computation. It just makes up for the shortcomings of the attention mechanism.

Liu et al. (2021b) proposed dual self-attention with co-attention networks (DSACA) for the purpose of capturing the internal dependencies between images and interrogatives and the intrinsic dependencies between different interrogatives and different images. It attempts to fully use the intrinsic correlation between question words and image regions in VQA to describe the internal dependencies of spatial and sequential structures independently using the newly developed self-attentive mechanism to effectively reason with answer correlations. The model is composed of three main submodules: a visual self-attention module to capture the spatial dependencies within images for learning visual representations; a textual self-attention module to integrate the association features between sentence words to selectively emphasize the correlations between interrogative words; and finally, a visual-textual co-attention module to capture the correlations between images and question representations. This approach successfully combines local features and global representation for better representation of text and images, which enormously works on the effectiveness of the model. Chen, Han & Wang (2020) proposed a Multimodal Encoder-Decoder Attention Network (MEDAN). The MEDAN consists of Multimodal Encoder-Decoder Attention (MEDA) layers cascaded in depth, and can capture rich and reasonable question features and image features by associating keywords in question with important object regions in image. Each MEDA layer contains an Encoder module modeling the self-attention of questions, as well as a Decoder module modeling the question-guided-attention and self-attention of images. Zheng et al. (2022a) and Zheng & Yin (2022) propose an SCF-DMN model that includes a self-attention mechanism, in which a model independent meta-learning algorithm was introduced and a multi-scale meta-relationship network was designed. adds the model-independent meta-learning algorithm and designs a multi-scale meta-relational network, as can be seen in Fig. 9.

Yu et al. (2019) proposed Modular Co-Attention Network (MCAN) to refine and understand both visual and textual content. Inspired by the Transformer model, the author sets two general attention units: a self-attention (SA) unit for modal internal interaction and a guided attention (GA) unit for modal interaction. Then a Modular Co-Attention layer is used to connect the two units in series. Finally, multiple module layers are connected in series. This co-attention mechanism, in the form of co-learning problems and images, can effectively reduce features that are not relevant to the target, exploit the correlation between multi-modal features, and can better capture the complex interactions between multi-modal features, which can improve the performance of VQA models to some extent.

However, behind the powerful ability of the self-attention mechanism, there is a drawback that cannot be ignored: when the model is encoding the information of the current position, it will focus excessively on its own position. To solve this problem, we can improve it by the method of multi-layer attention mechanism.

The multi-layer attention mechanism (Zheng, Liu & Yin, 2021), on the other hand, is an evolved version of the single-layer attention mechanism, which allows the model to distill information about the features in the problem from different dimensions by performing multiple operations. This mechanism permits the model to pay joint attention to information coming from various subspaces in various regions, an aspect that is not possible with other types of attention mechanisms.

As the difficulty of the problem increases, more and more VQA models use multiple attention levels to capture deeper visual language associations. Yu et al. (2017) proposed a multi-level attention mechanism, including Semantic Attention, Context-aware Visual Attention, and Joint Attention Learning. However, the negative consequences of more layers are parameter explosion and over fitting of the model. Inspired by the capsule network, Zhou et al. (2019) proposed a very compact alternative to achieve accurate and efficient attention modeling, called dynamic capsule attention (CapsAtt). CapsAtt regards visual features as capsules, obtains attention output through dynamic routing, and updates the attention weight by calculating the coupling coefficient between the bottom capsule and the output capsule.

**Figure 10:**
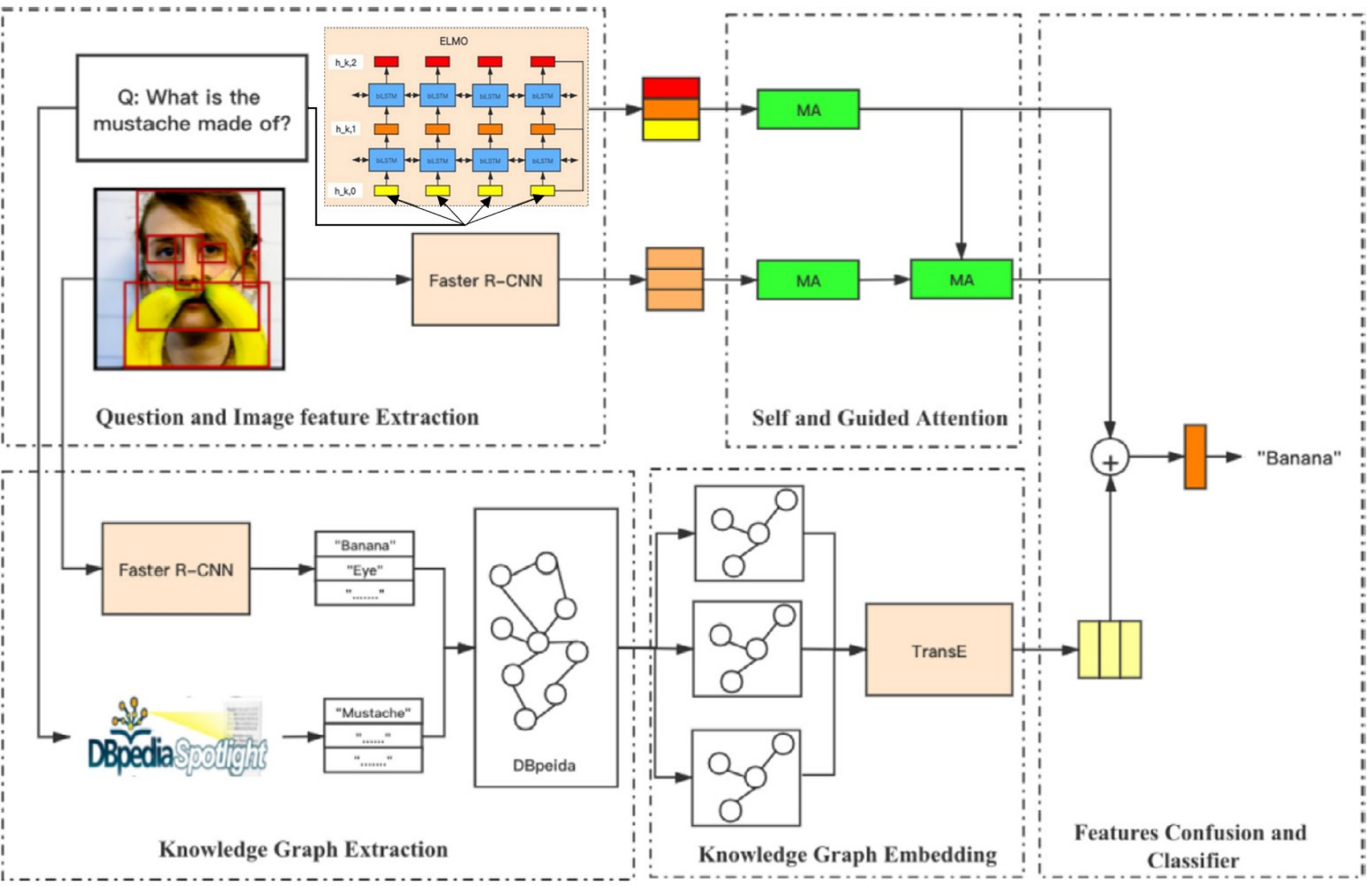
The infrastructure of N-KBSN model (Ma et al., 2021; Zheng et al., 2021).

Meanwhile, we found that some previous models overly rely on attention mechanisms to associate query words with image content to answer relevant questions. However, they are usually oversimplified to linear transformations, leading to erroneous correspondence between questions and visuals, poor generalization capacity, and possible limitations, and it may not be enough to fully capture the complexity of multi-modal data. In this context, reseachers introduced the method of adversarial learning (Liu et al., 2018; Liu et al., 2021a; Sarafianos et al., 2019; Wu et al., 2018b; Xu et al., 2018; Ilievski & Feng, 2017). Ilievski & Feng (2017) suggest an attention mechanism based on adversarial learning in this situation that generates more varied visual attention maps and improves the generalization of attention to new issues, leading to improved learning to capture complicated multi-modal data linkages. Liu et al. (2021a) proposed a framework based on adversarial learning to learn joint representation to effectively reflect information related to answers. Ma et al. (2021) proposed an N-KBSN model that introduced dynamic word vectors based on self-attention and guided attention-based multi-head attention mechanisms, and found that the accuracy of the results exceeded that of the winners (gloves) models of 2017 and 2019, Zheng et al. (2021) then introduced a higher-level representation named semantic representation and obtained a better result. As shown in Fig. 10.

## Discussion

VQA is the basic field for realizing artificial intelligence, and improving the efficiency and accuracy of models is a hot spot in current research, The attention mechanism is very important for achieving effect improvement in VQA (Zheng et al., 2022b; Patro, Anupriy & Namboodiri, 2022; Peng et al., 2022b). Therefore, we review the development status of V QA and find that the attention mechanism has received more and more attention from scientists, and it is widely used in VQA models and has multiple forms of existence.

We selected the models with the highest citation rates in each year from 2016 to 2022 for collation and comparison, and our findings are shown in Table 2.

**Table 2:** Comparison of the highest cited models in different years.

Model	Year	Free region	Object-Detection	Co-attention	Relation-attention	Self-attention
Ren et al. (2017)	2016	✓				
Ronneberger, Fischer & Brox (2015)	2016	✓		✓		
Bahdanau, Cho & Bengio (2015)	2016	✓				
Luong, Pham & Manning (2015)	2016		✓			
Anderson et al. (2018)	2017	✓		✓		
Chen et al. (2017)	2017	✓		✓		
Xu et al. (2015)	2017	✓				
Wu et al. (2017)	2017	✓		✓		
Antol et al. (2015)	2017	✓			✓	
Zhu et al. (2016)	2017	✓		✓	✓	
Zheng et al. (2020)	2018		✓			
Pan et al. (2022)	2018		✓	✓		
Li et al. (2022)	2018	✓		✓		
Gong et al. (2021)	2018	✓		✓	✓	✓
Zhan et al. (2020)	2018	✓				
Wang et al. (2022a)	2019		✓	✓	✓	✓
Wang et al. (2022b)	2019		✓	✓	✓(graph)	✓
Bazi et al. (2022)	2019		✓	✓	✓	✓
Zheng et al. (2022b)	2019	✓		✓	✓(graph)	
Al Rahhal et al. (2022)	2020	✓		✓	✓(graph)	
Gurari et al. (2018)	2020		✓	✓	✓	
Tung et al. (2021)	2020		✓		✓	
Tung, Huy Tien & Minh Le (2021)	2021		✓	✓		✓
Liu et al. (2019)	2021		✓	✓		
Gao et al. (2019)	2021	✓		✓	✓	
Selvaraju et al. (2020)	2021	✓		✓	✓	✓
Gao et al. (2015)	2022	✓	✓	✓		
Malinowski et al. (2015)	2022		✓	✓	✓(graph)	
Yang et al. (2019a)	2022	✓	✓	✓	✓	✓
Han et al. (2021)	2022		✓	✓	✓(graph)	
Yu et al. (2019)	2022		✓	✓	✓	✓

In Table 2, we have sorted out the attention-based model that were the mostly cited every year since 2016. The relational attention representation model pays attention to the relationship between different areas of the picture or different objects, and (graph) represents the model uses knowledge-based or factor-based graph attention. It can be seen that over time, researchers have proposed a variety of attention modules, and the complexity of the model has also increased.

For image attention, researchers initially focused on finding the image area related to the problem. Later, with the development of object detection technology, researchers also introduced object detection into the VQA task, so as to find the object concerned by the problem and the semantic relationship between different objects. For text attention, researchers put forward the view that problem attention and image attention are equally important. They used attention maps to rank each word in the question and select the most important word. The introduction of relational attention and self-attention also enables VQA tasks to more accurately understand the semantic relationship inter and intra modes. However, this is still far from enough for such a complex cross-modal task. In order to further understand the high-level semantic information in the image, such as attributes and visual relationship facts, researchers introduce a factor based priori condition or knowledge graph, encode each image into a graph, and learn the attention relationship between different nodes and edges. This also greatly improves the accuracy and interpretability of VQA tasks, which may become the direction of further development of future work.

## Conclusion and Expectation

This review provides a brief overview of the attention mechanism approach to the VQA tasks, describing its classification, shortcomings, and existing methods of improvement, and we also use bibliometric methods to comprehensively analyze the current state of the VQA field and reasonably predict the attention mechanisms’ possible future directions.

Through a comprehensive study of the literature, we can reasonably infer that with the growing demand for real-time applications in AI, real-time attention may become a hot issue for future research. At the same time, we find that emotional attention and cross-modal retrieval will also become a hot issue for research in the future. Finally, the attention mechanism can be stand-alone (Ramachandran et al., 2019) and take the lead, and in the future, it may be possible to perform attention mechanism in a larger area or globally, and it can also open up the relationship between various modalities, enhance the adaptability to various dynamic scenarios, meet the requirements of different scenarios, process data from multiple modalities, reduce the training cost of machine learning, and form a general “intelligent machine” model with multidimensional interaction. The method based on graph attention may become a research hotspot in the future.

We found that although the attention mechanism is improving, there are still some shortcomings, and it still needs continuous exploration and research on how to break through these problems. By exploring hard in these aspects, we can make the VQA task develop toward a more intelligent, more accurate, and more humane direction.

## Additional Information and Declarations

### Data Availability

The following information was supplied regarding data availability: This is a literature review.
